# Perfusion decellularization of a human limb: A novel platform for composite tissue engineering and reconstructive surgery

**DOI:** 10.1371/journal.pone.0191497

**Published:** 2018-01-19

**Authors:** Mattia Francesco Maria Gerli, Jacques Paul Guyette, Daniele Evangelista-Leite, Brian Burns Ghoshhajra, Harald Christian Ott

**Affiliations:** 1 Center for Regenerative Medicine, Massachusetts General Hospital, Boston, Massachusetts, United States of America; 2 Harvard Medical School, Boston, Massachusetts, United States of America; 3 Department of Radiology, Massachusetts General Hospital, Boston, Massachusetts, United States of America; 4 Division of Cardiology, Massachusetts General Hospital, Boston, Massachusetts, United States of America; 5 Division of Thoracic Surgery, Department of Surgery, Massachusetts General Hospital, Boston, Massachusetts, United States of America; 6 Harvard Stem Cell Institute, Boston, Massachusetts, United States of America; Politecnico di Milano, ITALY

## Abstract

Muscle and fasciocutaneous flaps taken from autologous donor sites are currently the most utilized approach for trauma repair, accounting annually for 4.5 million procedures in the US alone. However, the donor tissue size is limited and the complications related to these surgical techniques lead to morbidities, often involving the donor sites. Alternatively, recent reports indicated that extracellular matrix (ECM) scaffolds boost the regenerative potential of the injured site, as shown in a small cohort of volumetric muscle loss patients. Perfusion decellularization is a bioengineering technology that allows the generation of clinical-scale ECM scaffolds with preserved complex architecture and with an intact vascular template, from a variety of donor organs and tissues. We recently reported that this technology is amenable to generate full composite tissue scaffolds from rat and non-human primate limbs. Translating this platform to human extremities could substantially benefit soft tissue and volumetric muscle loss patients providing tissue- and species-specific grafts. In this proof-of-concept study, we show the successful generation a large-scale, acellular composite tissue scaffold from a full cadaveric human upper extremity. This construct retained its morphological architecture and perfusable vascular conduits. Histological and biochemical validation confirmed the successful removal of nuclear and cellular components, and highlighted the preservation of the native extracellular matrix components. Our results indicate that perfusion decellularization can be applied to produce human composite tissue acellular scaffolds. With its preserved structure and vascular template, these biocompatible constructs, could have significant advantages over the currently implanted matrices by means of nutrient distribution, size-scalability and immunological response.

## Introduction

Perfusion decellularization is an established bioengineering technology allowing for the generation of extracellular matrix (ECM) scaffolds from donor organs and tissues, via circulation of detergents through the native vasculature [1]. While other decellularization techniques are based on passive diffusion or physical insults, perfusion decellularization utilizes the native vascular tree to distribute detergents, providing better access, deep tissue exposure, and improved removal of the cellular components from large three-dimensional tissue compartments [1–3]. In the past decade, this technology has been utilized to bioengineer acellular scaffolds from donor human lungs, hearts, kidneys, livers, and pancreases not suitable for transplantation [4–8]. The acellular vascular network has been successfully repopulated with patient-specific endothelial cells and pericytes. This enables graft anastomoses with the recipient circulation as recently reported for bioartificial human and porcine lungs [9, 10]. In 2015, our group reported for the first time the use of this technology for the production of acellular composite tissue scaffolds from whole rat and non-human primate extremities [11]. The work demonstrated successful removal of cellular components and preservation of essential ECM proteins across all tissue components of the limbs.

Translating these findings into human composite tissues would have an important impact in the field of reconstructive surgery, for patients affected by soft tissue loss and at risk of amputation. Soft tissue and volumetric muscle loss (VML) is the frequent outcome of trauma-repair surgeries, solid tumour resection, exposed bone fractures, burns, combat injuries and diabetes complications, accounting in the US, for around 5.8 million procedures per year [12]. VML patients face the challenging psychological consequences of a relevant loss in tissue mass, muscle strength and function, often leading to a permanent disability [13]. Muscle and fasciocutaneous flaps, taken from autologous donor sites are currently the most utilized approach for VML and limb trauma repair [14, 15]. In the case of skin grafting, a portion of autologous tissue can be surgically transposed to the injured site without its nourishing blood supply (free-grafting), significantly improving the healing process at the implanted site [16, 17]. However, the volume and shape of the available donor tissue is limited. Outcomes are further reduced by the inevitable morbidity due to tissue loss at the donor site, and complications arising from having two surgical sites [18, 19]. As a consequence of these limited donor tissue options, the volume of the soft tissue injury or defect has been shown to largely impact the clinical discernment regarding limb salvage versus amputation [20].

Cell-based human skeletal muscle engineering aims to generate bio-artificial muscle tissue *in vitro*, in order to provide replacement material and compensate for tissue loss [21–24]. While various approaches may soon find potential application for *in vitro* disease modelling and drug discovery, none have yet to succeed in generating human contractile grafts on a clinically relevant scale [25, 26].

Although the long-term consequences of trauma-related soft tissue loss are disabling, resulting functional defects are non-life threatening. Therefore, the balance between risk and benefit to any novel therapeutic approach requires careful judgement. Hence, implementing simpler but effective tissue engineering approaches, such as the creation of directly implantable, tissue-specific acellular matrices, might have a relevant clinical impact for soft tissue loss in the near future. Acellular matrices foster a broad spectrum of tissue engineering applications with at least two dozen ECM products currently being tested in humans for dermal and reconstructive surgery applications [27]. The primary objective of VML implants is to compensate for lost tissue volume, promoting cellular ingrowth and improving muscle function through direct force generation. Recent reports on VML patients indicate that it is possible to meet some of these milestones using bare xenogeneic acellular matrices derived from porcine skin, intestine, and bladder. These three-dimensional ECM scaffolds have been shown to boost the regenerative potential at the injured site. This was shown in two small cohorts of trauma-related VML patients (n = 1 and n = 5, respectively), triggering the endogenous tissue repair process and providing an environment that promotes vascular and perivascular cell migration [28, 29]. Biopsies taken from implant sites showed successful vascular ingrowth but limited evidence of *de novo* myogenesis. A small number of newly formed myofibres were observed, mainly located at the intersection with the native tissue. Functional enhancement achieved in these pivotal studies may have been due to alternative means of strength improvements, such as functional fibrosis, or as a result of the strong physical therapy regime applied, rather than to direct muscle regeneration [30, 31]. A follow up study on a larger cohort of patients (n = 13, 5 of which included as a follow-up from the Sicari’s study), provided solid evidence that patients showed improved direct force generation, electrophysiological function and task completion. In addition, this study reported extensive ECM remodelling, showing improved vascularization and the appearance of regenerating myofibres within the implant [32]. Interestingly, it is possible that the promising regenerative performance highlighted by these studies could have even been limited by the use of non tissue-specific xenogeneic scaffolds [33]. Thanks to our previous experience in decellularizing composite tissues, we aimed at producing the first acellular human limb, as a potential source of full-size allogeneic grafting material. By matching more closely the ECM composition and tissue architecture to the native tissue, this construct could provide a more physiological foundation for tissue regeneration at the grafted site.

The long-term goal of this project is the production of composite tissue scaffolds with preserved three-dimensional architecture, enabling improved integration and vascularity, thus sustaining the oxygen and nutrient demands necessary for regenerating tissue. In this proof-of-concept study, we therefore evaluated the perfusion decellularization of a complete cadaveric human upper extremity with the aim of creating an anatomically preserved, clinical-scale construct comprising all the tissue compartments of the native limb. A human, perfusable, tissue-specific, composite scaffold could provide significant advantages to the healing process, contributing to tissue repair by improving nutrient distribution and graft integration. If successful, the resulting tissue matrices could not only be applied to soft tissues in reconstructive surgery, but also for skeletal muscle augmentation, in contribution to the growing fields of limb bioengineering and bio-prosthetics.

## Materials and methods

### Experimental design

The objective of the study was to investigate the possibility of generating a perfusable acellular scaffold from a full human cadaveric extremity for possible future tissue engineering applications. An anonymized cadaveric human upper extremity was obtained from a 57 years old male donor through the National Disease Research Interchange (MGH IRB Protocol #: 2011-P-002433/1). Based on the previous non-human primate study we requested that the limb was explanted within 24 hours post-mortem and snap frozen for shipment. The inclusion criteria for the procurement required: patients with BMI < 30, no diagnosis of Diabetes, infectious disease or recent chemotherapy treatment. We excluded CDC high-risk categories donor. The specimen was tested for HIV, Hepatitis B and C prior shipment clearance.

### Construction of a bioreactor for the decellularization of a full human limb

**Fig 1:**
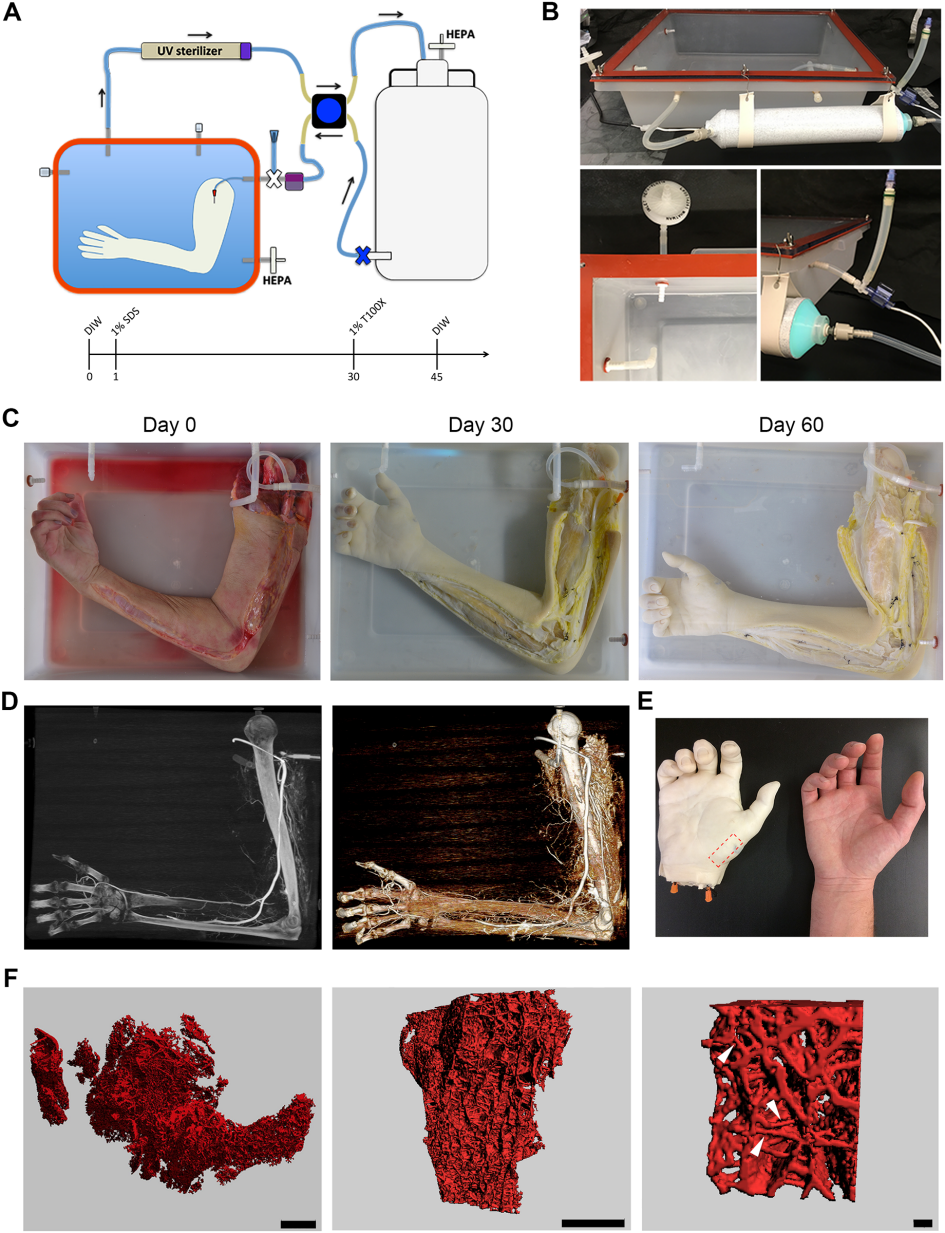
Perfusion decellularization of a human upper extremity. **(A)** The scheme depicts the bioreactor designed to allocate and decellularize a full human limb and the detergent perfusion timeline utilized for the experiments presented in the article. **(B)** The panel presents highlights on the main components of the bioreactor: the perfusion chamber (top), HEPA filters, pressure sensor and UV sterilizer (bottom). **(C)** Images of the human arm at the beginning of the procedure, after 30 days in perfusion with SDS and at the end of the decellularization procedure. **(D)** Computer tomography images showing the perfusion of contrast fluid in the brachial, ulnar and radial artery and palmar arch (left), as well as in the medium size vessels of the proximal arm. **(E)** Macroscopic comparison of the decellularized hand with a native control. The orange 15 gauge stubs have been subsequently utilized to perform Microfil silicon casting. The red rectangle indicates the region of the *Abductor Pollicis Brevis* muscle explanted and utilized for the μCT scan experiments. **(F)** 3D reconstructions of μCT scans obtained from the *Abductor Pollicis Brevis* muscle depicting the preserved microvascular architecture at different magnifications (scale bars: 1 mm, 1 mm, 100 μm).

Building up on the results obtained with the bioreactor utilized for non-human primate forearms, we designed and built a perfusion decellularization chamber for full human extremities (Fig 1a). Our decellularization chamber is based on a polypropylene autoclavable tray (Nalgene; 6900–0020). We equipped the bioreactor with 50 litres fluid reservoirs (Nalgene; 2319–130) and used autoclavable PVDF connectors and low resistance silicone tubing (Cole Parmer). We sealed the top of the chamber using a custom-made silicon gasket and a polycarbonate lid that was secured to the bioreactor through six stainless-steel bolts (McMaster-Carr; 86045K23, 8574K55). To secure ventilation while maintaining sterility, both chamber and solution reservoirs were equipped with HEPA filters (Whatman; L#9514261) and an in-line UV sterilizer was introduced in the system to further lower the bioburden. The bioreactor was autoclaved and all the procedures have been carried out in a laminar flow hood. Fluid perfusion was applied to the organ through the brachial artery through a 4-roller computer-controlled peristaltic pump (Masterflex—EasyLoad II). We monitored the perfusion pressure through a disposable in-line pressure sensor (Pendotec—PressureMAT; Fig 1b).

### Perfusion decellularization of a full human upper extremity

The limb was let thaw overnight at 4°C, washed and prepared for sterile dissection. We cannulated the brachial artery with a 15 gauge blunt needle as access for solution perfusion. The brachial vein was also cannulated to allow monitoring of the vascular outflow. As previously described for non-human primate extremities, we performed bilateral fasciotomies before initiating the detergent perfusion. This is done to allow the radial tissue expansion due to the changes in the osmotic charge during the process and avoid the occurrence of a comportment syndrome (Fig 1c). We first flushed the specimen with 6 litres of heparizined saline solution to remove blood and blood clots from the vasculature. We then perfused the limb with two changes of 40 litres of autoclaved deionized water single-passage at 60 ml / minute. We subsequently perfused 1% v/w Sodium Dodecyl Sulphate (SDS) for 30 days and 1% Triton-X (Sigma Aldrich; T8787) for 15 days in a closed loop recirculation system with a 50 litres reservoir replacing the detergents solution every other day. Perfusion speed was started at 60 ml / min and incremented up to 120 ml / min with the proceeding of the decellularization. During the procedure, we tied off the vascular leaks caused while operating the fasciotomies using 2–0 sutures. To avoid clogging of the perfusion system, we introduced an in-line 100 μm nylon mesh (McMaster-Carr; 3802T511) on the return line. The bioreactor was closely monitored for the presence of floating epidermis fragments detaching from the specimen, which have been manually removed with an aspirator. At the end of the decellularization procedure, autoclaved deionized water was perfused in the organ for 15 days changing the 50 litres reservoir every 12 hours to allow the removal of the detergents from the matrix. The organ was then re-equilibrated through perfusion of 50 litres of sterile phosphate buffer solution (PBS) and used for histological and biochemical validation.

### CT and μCT scans

Computer tomography scans were performed through a dual-source CT scanner (SOMATOM Definition Flash, Siemens Healthcare). The perfusion bioreactor was positioned on the scanning table and the limb was perfused with a 1:1 mix of PBS and Iohexol 350 mg / ml (GE Healthcare; Omnipaque) contrast solution injected at 2 ml / second. Scanning was performed using a multiphase volume dynamic protocol, acquiring five consecutive scans 2 seconds apart with a section thickness of 0.6mm. Dynamic 3D reconstructions (volume rendered, mutiplanar reformatted images, and maximum intensity projection views) were performed using Osirix MD (Pixmeo, Switzerland). For μCT scan, silicon casting was performed using a radiopaque red Microfil kit following the manufacturer procedures (Flow Tech, MV-130 Red). Briefly, the hand was isolated from the decellularized limb and both radial an ulnar artery have been cannulated. 120 millilitres of Microfil solution were perfused through the radial artery while increasing the perfusion pressure by clamping the ulnar artery. The cast was cured overnight at 4°C before proceeding with the biopsies. μCT scan, image analysis and 3D reconstruction were performed through a Scanco Medical μCT40 at the MGH Skeletal Core Imaging Facility with section thickness of 6μm to allow resolving small calibre vessels.

### Histological and immunofluorescent staining

Tissue samples were fixed overnight in 2% paraformaldehyde at 4°C and subsequently processed for paraffin embedding. Bone samples have been decalcified through 20% EDTA prior embedding. 7 μm thick sections were obtained through a Leica RM2155 microtome. The slides have been deparaffinized, rehydrated and subjected to the following staining: Hematoxilin and Eosine (Sigma Aldrich); Masson’s Trichrome (American Master Tech); Verhoeff’s elastic stain (American Master Tech). For immunofluorescent analysis, we performed antigen retrieval on deparaffinized sections using a citrate-based antigen unmasking solution (Vector Laboratories; H-3300). The sections were blocked with 1%BSA and 1% Donkey Serum (Sigma Aldrich) and incubated overnight at 4 C with the following primary antibodies: mouse anti-myosin (DSHB; MF20); mouse anti Sarcomeric alpha-actinin (Sigma Aldrich; SAA A7811); rabbit anti Laminin (Abcam; ab11575); rabbit anti Collagen IV (Abcam; ab6586); mouse anti Neurofilament H (Sigma Aldrich, NF200 ab5539); rabbit anti S100 calcium binding protein B (Abcam; ab52642). After primary antibody binding, the sections were washed and incubated for one hour at room temperature with the appropriate donkey-raised secondary antibodies, conjugated with a 488, 546 or 647 fluorochromes (Life Technologies; AlexaFluor series).

### Biochemical analyses

Nuclease treatment was performed by treating native and acellular biopsies with Pierce Universal Nuclease (Thermo Fisher: 88701) at a concentration of 25 U / ml for 24 hours at room temperature. DNA extraction was performed using the Qiagen DNeasy blood and tissue kit and the double strand DNA concentration was assessed with the Quant-iT PicoGreen dsDNA assay (Life Technologies; P7589) following the manufacturer protocols. Fluorescence was read at 480nm/520nm (excitation/emission) and concentrations were calculated in relation to the standard curve. All the proteomic analysis have been performed using a Spectra Max M5 plate reader (Molecular devices) on a minimum of 3 independent biopsies taken from different areas of the specimen, and reported in the graph as average readings. Collagens were quantified using the Sircol soluble and insoluble collagen assay kit (Biocolor) following the standard manufacturer procedures. Briefly, lyophilized tissue was incubated overnight at 4°C in 500uL of 0.2 mg / ml of pepsin in 0.5 M acetic acid. For soluble collagen analysis, the acid/pepsin solution was concentrated as instructed in the manual. The extract was incubated with Sircol Dye Reagent for 30min followed by ice-cold Acid-Salt Precipitation Reagent. For insoluble collagen analysis, the residue remaining after the acid/pepsin digestion was incubated in Fragmentation Reagent (50 ul / mg) for 3h at 65°C, and centrifuged at 12,000rpm for 10 min. Samples were then incubated with Sircol Dye Reagent for 30min, washed with 750 μl of ice-cold Acid-Salt Precipitation Reagent, centrifuged and decanted. The final pellets were solubilized with 250 μl of Alkali Reagent and absorbance was measured at 555nm and concentrations were calculated in reference to the relative standard curve. Elastin was quantified using the Fastin Elastin assay (Biocolor) as instructed in the manual. Briefly, lyophilized tissue (~10 mg dry weight) was digested three times for 1h at 60°C in 750 μl of 0.25 M oxalic acid. Elastin was precipitated and pelleted at 14,000g for 10min. The pellet was then stained using 1 ml of dye reagent for 90min at room temperature, centrifuged and re-suspended in 250μl of dye dissociation reagent for 10 min. Absorbance was read at 513nm and compared to a standard curve to calculate the final elastin concentration. Glycosaminoglycans (GAGs) analysis was performed using the Blyscan kit (Biocolor). Dry samples were mechanically homogenized in 1 ml of papain buffer containing 7 μl / ml of papain, digested for 4h at 65°C and incubated with the dye reagent. After washing, the samples were read in a 96-well plate, at 656nm and concentrations were calculated in reference to the relative standard curve.

## Results

### Generation of an intact decellularized human upper extremity with preserved perfusable vascular architecture

We obtained an anonymized cadaveric human arm from an adult male donor, through the National Disease Research Interchange (MGH IRB Protocol #: 2011-P-002433/1). After cannulation of the brachial artery, we performed bilateral fasciotomies, positioned the specimen in a custom-designed bioreactor, and started perfusion of rinsing and decellularizing solutions (A schematic of the bioreactor and of the experimental timeline is reported in Fig 1A and 1B; see Material and methods for further details). Based on the experience acquired with non-human primate limbs, we chose a detergent perfusion speed of 60–120 ml / minute, lower than the brachial artery physiological flow (normally 6–800 ml / minute) to reduce the potential development of compartment syndrome. We non-invasively evaluated the progression of the decellularization by visually monitoring the colour change of the specimen, starting from the red-pink characteristic of the native muscle tissue, to the white / yellow tone typical of the acellular matrix scaffolds (Fig 1C). Contrast-enhanced computerized tomography scans (SOMATOM Definition Flash, Siemens Healthcare) performed using overlapping 0.6 mm thick sections, confirmed that the macroscopic vascular architecture of the limb was preserved and patency was maintained showing distal perfusion up to the palmar arches (Fig 1D; S1 Movie). We subsequently isolated and cannulated the decellularized hand (Fig 1E) and performed a Microfil silicone casting with the aim of resolving the hierarchical structure and network of the microvasculature. A series of high-resolution μCT scans were performed on muscle biopsies taken from the *Abductor Pollicis Brevis*, using a Scanco Medical μCT40 scanner set to take 6 μm sections for acquisition. This allowed resolving the vascular network up to venules and arterioles, confirming successful perfusion and preservation of the small vessels in the decellularized hand (Fig 1F, white arrowheads).

### Histological analysis of the tissue compartments

**Fig 2:**
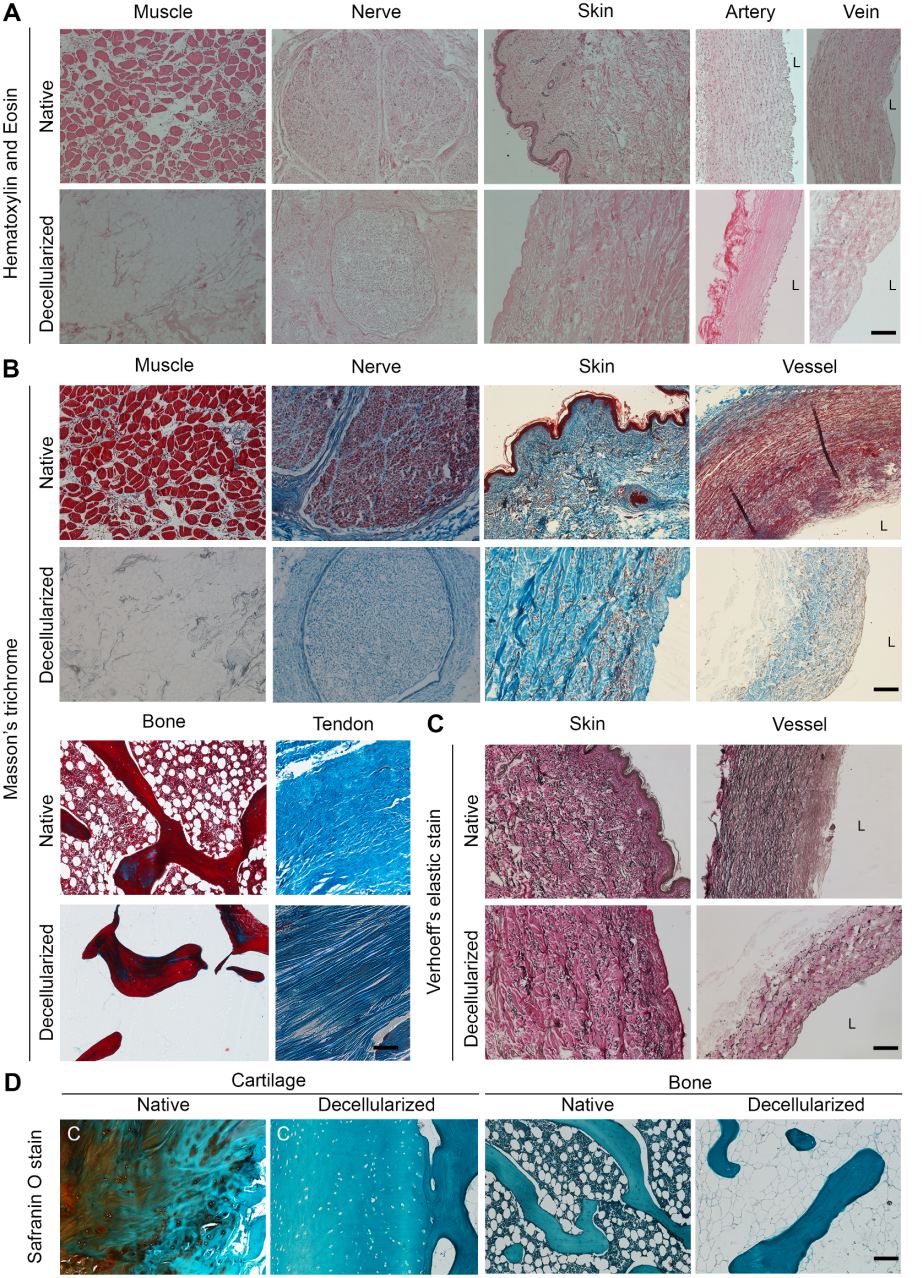
Histological characterization of the tissue composing the decellularized limb. **(A)** The panel shows bright field images of the Haematoxylin and eosin staining performed on muscle, nerve, skin and vessels before and after the decellularization procedure, highlighting absence of nuclei and reduction in the eosin positive structures across the tissues (L indicates the vascular lumen; scale bar: 100 μm) **(B)** The panel shows bright field images of a Masson’s trichrome staining performed on muscle, nerve, skin, vessels bone and tendon, highlighting the removal of the nuclei and cellular components such as sarcomeres, axons, epidermis, smooth muscle and bone marrow (black and red) and preservation of the collagenous matrix proteins (blue; L indicates the vascular lumen; scale bar: 100 μm). **(C)** Phase contrast images of a Verhoeff’s elastin stain indicating the preservation of the elastic fibres (black) in section of native and decellularized skin and vessels (L indicates the vascular lumen; scale bar: 100 μm). **(D)** Bright field microscopy images of a Safranin O / Fast green stain performed on native and decellularized cartilage and bone tissue highlighting removal of the proteoglycans (orange), bone marrow (blue) and nuclei (black; scale bar: 100 μm).

To validate the successful decellularization, a series of tissue biopsies from different compartments of the acellular limb scaffold were harvested, and then examined for content and structure by histological staining. Haematoxylin and Eosin staining confirmed the successful removal of the nuclei from the muscle, nerve, skin, and vascular compartments (Fig 2A). Moreover, a drastic reduction in the dense eosin-positive areas across the muscle and skin provided initial indication of successful decellularization. A Masson’s trichrome staining indicated the red Fuscin-positive intracellular structures, such as the muscle sarcomeres, the axons in peripheral nerve the epidermis, and the vascular smooth muscle were successfully removed from the tissues upon decellularization (Fig 2B). In addition, we observed that the blue Aniline-positive collagenous structures were largely preserved across the tissues, which retained its intact three-dimensional matrix structure. Furthermore, the Fuscin / Haematoxylin-positive bone marrow was successfully removed from the trabecular bone, while the bony structure was preserved. The nuclei were successfully removed from the tendons while the aniline-positive collagenous fibres were preserved (Fig 2B). Verhoeff’s elastic stain allowed us to observe the preservation of the elastic fibres in skin and vessels biopsies. These fibres are a crucial functional component of these tissues, being responsible for its elasticity (Black; Fig 2C). Lastly, we performed a Safranin O / Fast green stain on cartilage and trabecular bone sections. This staining indicated a reduction in the proteoglycan content (orange) of the cartilage, confirmed removal of the bone marrow from the trabecular bone, and highlighted the elimination of the nuclei (black) from both the tissues (Fig 2D).

### Validation of cellular component removal and ECM protein preservation via immunofluorescent staining

**Fig 3:**
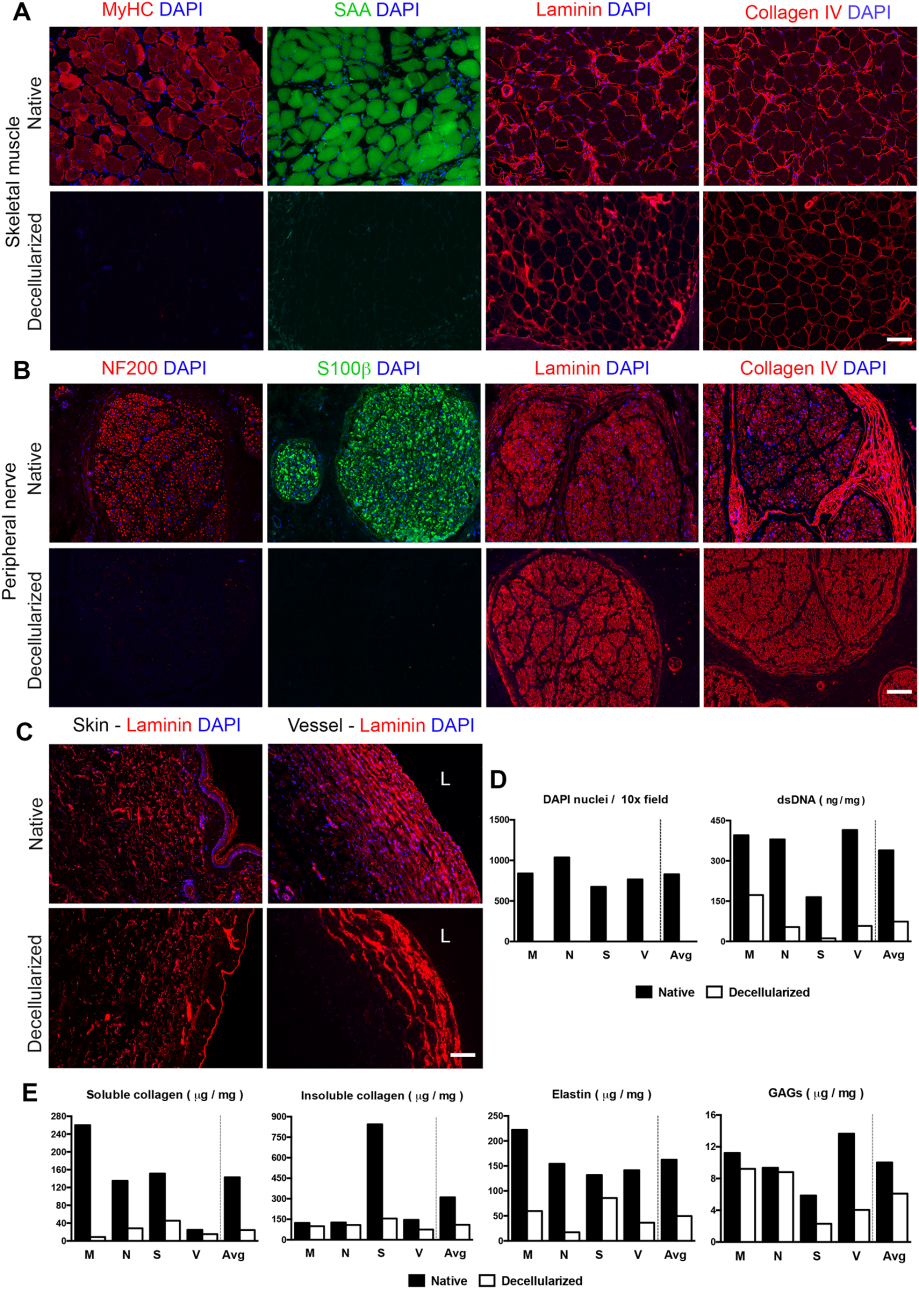
Molecular characterization of the different compartments of the acellular scaffold. **(A)** The immunofluorescence images depict native and acellular muscle sections stained for the sarcomeric proteins Myosin (MyHC) and Alpha-Actinin (SAA) and for the extracellular matrix proteins Laminin and Collagen IV. Sections were counterstained with DAPI to confirm the removal of the cell nuclei upon decellularization (scale bar: 100 μm). **(B)** Immunofluorescent imaging of native and acellular peripheral nerve sections stained for the axon protein Neurofilaments (NF200), the Schwann’s cell marker S100β and for the extracellular matrix proteins Laminin and Collagen IV. Sections were counterstained with DAPI to confirm the removal of the cell nuclei upon decellularization (scale bar: 100 μm). **(C)** Immunofluorescent staining for Laminin on skin and vessel sections (L indicated the vessel lumen; scale bar: 100 μm) **(D)** Quantification of the number of DAPI-positive nuclei and residual double strand DNA concentration across native and decellularized nerve (N), muscle (M), skin (S) and vessel (V) biopsies (left). Quantification was performed using a PicoGreen fluorescent assay (measures are presented as: ng of dsDNA / mg of dry tissue). **(E)** The bar graphs indicate: the concentration of soluble and insoluble collagen measured via Sircol assay; the Elastin concentration measured via Fastin fluorescent assay; the GAGs concentration quantified via Blyscan fluorescent assay across three independent muscle (M), nerve (N), skin (S) and vessel (V) biopsies (measures are presented as: μg of protein / mg of dry tissue).

To confirm the removal of intracellular components and preservation of extracellular matrix components at the protein level, we performed a panel of immunofluorescent staining on the main tissue composing the limb. As expected, myosin heavy chain (MyHC) and sarcomeric alpha actinin (SAA), crucial components of the skeletal muscle contractile apparatus, were fully removed from the muscle tissue (Fig 3A). We observed instead that Laminin and collagen IV, main components of the muscle ECM, were preserved through the decellularization process. Similarly to our observation in muscle samples, sections of peripheral nerve staining confirmed the successful removal of the axons neurofilaments (NF200) and of the S100 calcium binding protein B (S100β) that typically marks glial cells (Fig 3B). Laminin and collagen IV, which naturally ensheathes the peripheral nerve bundles and the axons, were preserved through the decellularization process, similarly to what observed for the muscle matrix (Fig 3B). As observed in muscle and peripheral nerve tissue stains, laminin was preserved also in elastic tissues like skin and vessels (Fig 3C). Overall, we observed successful removal of the nuclei by DAPI-positive counting, from all the tissue types analysed (Fig 3A, 3B and 3C; Quantification in Fig 3D).

### Quantitative assessment of residual DNA and matrix composition

With the aim of reducing its residual DNA content, we subjected the acellular specimen to a nuclease treatment. We then performed a PicoGreen assay to quantify the amount of double strand DNA left in the matrix. Consistently with the removal of the nuclei observed through histological and DAPI stain, the dsDNA assay indicated an average 4.6-fold reduction in the double strand DNA content across the various tissues composing the matrix (Native 333.8 ± 116.8 ng / mg; Decellularized 73.82 ± 69.19 ng / mg; Fig 3D). To investigate the matrix composition at the molecular level we performed a panel of biochemical assays aiming to quantify the residual amounts soluble and insoluble collagen, elastin and glycosaminoglycans from biopsies harvested from different limb compartments taken before and after and decellularization. To compare the soluble and insoluble collagen concentration in native and decellularized tissues, we performed a Sircol quantitative assay on lyophilised biopsies. Our results indicated an average 4.8-fold decrease in the concentration soluble collagen across the tissues (Native 142.8± 96.4; Decellularized 24.4 ± 16.1 μg / mg of dry tissue), with the highest reduction observed in the muscle tissue. In contrast, we observed better preservation of the insoluble collagens in the decellularized matrix, with an average reduction of 1.8-fold (Native 310.1 ± 356.3 μg / mg; Decellularized 109.4 ± 33.74 μg / mg of dry tissue). Through a Fastin quantitative assay, we also observed a 2.3-fold reduction in the elastin content across the analysed tissues (Native 162.4 ± 49.8 μg / mg; Decellularized 49.8 ± 29.6 μg / mg). We then used a Blyscan assay, to evaluate the amount of residual glycosaminoglycans (GAGs) in the acellular matrix. When compared to the native tissues GAGs, result to be generally preserved by the process, showing only a 0.6-fold decrease (Native 10 ± 3.3; Decellularized 6 ± 3.5). Notably, all the matrix components analysed were still present at detectable levels across the tissues.

## Discussion

The current study successfully demonstrates the creation of a human-scale acellular limb scaffold, by augmenting the process of perfusion decellularization, and therefore highlighting the utility of this methodology for its use on full human extremities. A whole human arm was subjected to detergents perfusion via the native vasculature. Effective solubilization and removal of intracellular components (*e*.*g*. nuclei and contractile apparatus proteins) was observed across the major tissue compartments of the limb: muscle, nerve, skin, vessels, tendon, cartilage and bone. The main ECM components including soluble and insoluble collagens, laminin, elastin and GAGs were preserved at detectable levels following the decellularization process. We demonstrated this both by immunofluorescent and biochemical assays, consistent with what has been previously observed in rat and non-human primate limb decellularization [11]. To the best of our knowledge, this is the first report on the generation of a complete composite tissue scaffold from a human limb, with preserved structural architecture and vascular template. The development of this platform represents the first successful milestone towards the longer-term goal of constructing functional, vascularized, bioartificial composite grafts for surgical reconstruction and muscle augmentation.

We detected successful removal of the nuclei from all the tissue analysed. A diffusion nuclease treatment was applied to the biopsies, to approach the 50 ng / mg threshold suggested as the gold standard for decellularized tissues [34]. Overall we observed an average 4.6-fold reduction in the double stranded DNA content across the decellularized tissue. Looking at individual tissue compartments, we detected higher degrees of dsDNA removal from tissue that received a higher detergent exposure such as the skin, vessels, and nerves. However, our results indicate that further improvements will be required to reach the desired threshold for the muscle tissue. As previously reported for the cardiac muscle, significant improvements in this direction could be achieved by perfusing the nuclease through the vasculature of the entire organ, at the end of the decellularization step, instead of operating the treatment by diffusion, on isolated biopsies [5]. Notably, some of the commercially available ECM products utilized for clinical dermal and soft tissue repair have also been reported to exceed these specifications [35, 36].

The main limitation of this study is that our observations are limited to a single specimen. This is due to the complexity of the procurement of fresh donor human limbs. Further studies on a larger cohort of donors will be required to refine the decellularization process and investigate questions such as the possible inter-individual variable response to the decellularization process (i.e. due to anatomical differences, for instance in specimens with greater or lesser muscle mass). However, as in the case of hand transplantation, donor families view the request to donate a part of a limb quite differently than, for example, the request for donation of a heart [37]. Internal organs do not elicit the same visual effects, therefore garnering greater donation rates. From donor’s relative perspective, a unique aspect to limb donation is that the physical integrity of the body is visibly altered in what may seem a startling way [38], significantly reducing specimen availability.

The current protocol requires two months to achieve complete decellularization of a full human extremity. This long-term exposure to detergents may have a detrimental effect on the biocompatibility of the ECM scaffolds [39, 40]. Further investigation on a larger number of specimens would allow refinement of the decellularization approach, increasing speed and efficiency of the process, with the overall aim of reducing detergent exposure. While bilateral fasciotomy is performed to alleviate the occurrence of compartment syndrome due to the expected swelling of the decellularizing tissue, the development of a more conservative surgical approach could for example reduce the superficial vasculature damages, reducing leakage that would likely speed up the decellularization process. In addition, the chosen perfusion speed of 60–120 ml / minute, was far below the 6–800 ml / minute physiologically observed in the brachial artery [41]. Follow-up studies will investigate the use of higher perfusion rates to increase vasculature recruitment, to facilitate the removal of cellular components, and to achieve faster decellularization with shorter detergents exposure. Similarly to that reported for hearts and lungs, we hypothesize that the decellularization procedure could be improved by keeping the specimen in suspension [5, 42]. This allows relieving the pressure generated by the weight of the tissue ultimately contributing to enhance perfusion. With the aim of reducing the matrix exposure to strong detergents (i.e. SDS), follow up studies may also investigate the possibility of using of milder detergents (i.e. Sodium deoxycholate) and intermittent decellularization cycles, similarly to what is used for detergent-enzymatic decellularization (DET) [43].

The generation of an off-the-shelf, human composite tissue scaffold could be advantageous in providing reconstructive surgeons with a variety of size-matched tissue-specific grafting material to rapidly repair damaged extremities. It is not unconceivable that as recently reported for acellular oesophageal scaffolds [44], these constructs could be generated, cryopreserved and made available on demand to match patient-specific reconstructive needs. Recent articles indicated that perfusion-decellularized abdominal muscles could be applied for the correction of large abdominal wall defects, as shown in rat surgical models [45, 46]. However, very limited evidence is available on the generation and use of native human acellular skeletal muscle scaffolds for tissue repair. Small muscle biopsies taken from cadavers and amputated limbs have been decellularized via diffusion and implanted in a surgical model of rabbit abdominal-wall defect, showing colonization by fibroblasts and vascular cells [47]. In light of these results, perfusion decellularization of human cadaveric limbs would have highly relevant implications to compensate for soft tissue loss, providing large quantities of native, species-specific regenerative material with a vascular network. This is further supported by what has already been achieved with xenogeneic matrices implantation in VML patients [28, 29, 32]. Interestingly, perfusion decellularization has been recently employed to generate full thickness acellular scaffolds for cadaveric human faces [48]. This further confirms the interest of the field in the creation of composite tissue allografts. The use of tissue-specific matrix matching could be crucial for triggering an innate regenerative response across the various tissue compartments. Indeed, tissue-specific matrices have been proposed to be superior in promoting tissue regeneration over the previously utilized non-specific scaffolds (i.e. intestinal sub-mucosa and bladder-derived matrices) [49]. When implanted soon after the injury, tissue-specific matrices could provide the spatiotemporal and biochemical signals required by the regenerating tissue, to promote integration at the damaged site and host progenitors differentiation [33, 50]. Moreover, preservation of ECM proteins may also play a crucial role in the maintenance of mechanical properties required for grafting [51]. Follow-up *in vivo* studies on our tissue- and species-specific perfusable matrices will aim to investigate their regenerative potential and immune-compatibility, together with their safety for use in potential future translational applications. Using large-animal models of surgical defects will allow us to investigate the use of this technology for reconstructive surgery and muscle augmentation. Conjugating growth factors as chemo-attractants to the scaffolds could potentially facilitate the mobilization of the endogenous progenitors to promote faster and effective repopulation *in vivo* [52, 53].

In addition to direct implantation, these acellular scaffolds could also be subjected to cell-based engineering *in vitro*. Naturally occurring scaffolds could provide the micro-environmental, chemical and biomechanical cues necessary to support cell attachment, migration and differentiation. Implementation of these processes post-decellularization would allow the constructs to be repopulated *in vitro* before transplantation. In this direction, our group is already implementing biomimetic organ culture systems for lungs, kidney, heart and bowel engineering [5, 6, 10, 54]. In addition, in line with our previous work in culturing rodent recellularized forearms [35] a recent report intriguingly indicated that native human forearms could survive to 24 hours of ex-vivo perfusion [55]. This provides further evidence of the feasibility of the perfusion culturing bioengineered human composite tissue.

While a clinical-sized bioengineered composite tissue graft cannot survive without a blood supply [56], our perfusion-based bioengineering platform produces scaffolds with a vascular template. We recently reported that the vasculature of decellularized human lungs could be repopulated *ex vivo* using endothelial and perivascular cells with a high degree of cellular coverage [9]. In addition, our group has already provided preliminary indications on the feasibility of the vasculature re-endothelialization in rodent and porcine composite tissue scaffolds [11, 35]. However, the largest component of our scaffolds is the skeletal muscle, with its contractile fibres and tight innervation. Different approaches could be utilised to achieve repopulation of this complex tissue: as reported for rat extremities myoblasts could be delivered in situ through multiple injection [11]. This process could be standardized using devices such as the needle matrix, currently under development for the treatment of muscular dystrophy patients [57]. As an alternative, non-canonical myogenic progenitors such as mesoangioblasts, capable of crossing the vessel wall, could be delivered through the graft vasculature, allowing broader cell distribution, similarly to what was achieved in dystrophic animal models and in a recent Phase I/II clinical trial [58–60]. Due to its similarities with the blood vessels structure, the engineering of the lymphatic system is likely feasible, although extensive study on the tissue engineering of this system are still needed [61]. A seminal paper from the Reichmann group provided proof-of-principle evidence on the use of lymphatic endothelial cells (LECs), to engineer functional lymphatic vessels in hydrogel-based skin implants [62]. It is not unconceivable that LECs could be utilized in the future to recellularize the lymphatic compartment of the acellular scaffolds. Further studies, will aim to develop these recellularization methodologies identifying the ideal cell types, origins (*e*.*g*. primary culture, adult stem cell derived, iPS cell derived, etc.), delivery procedures and establish “multicellular” culture media or regimens needed to achieve co-culture of multiple cell types coexisting in a complex tissue.

Here we show that it is possible to generate large amounts of native acellular matrix from snap frozen human cadaveric extremities harvested within 24 hours from the time of death. Donor hearts and lungs often fail to meet the criteria for transplantation, making these organs easily available for research purposes. Contrarily, due to the lower occurrence of composite tissue transplants and to our tight inclusion criteria, we experienced significant difficulties in obtaining freshly explanted donor extremities for over two years. As new modalities in regenerative medicine garner attention and use, the field is hopeful that tissue and organ donors see increased value in extremity donation, as well. Interestingly for our study, previous observation did not find relevant differences with matrices derived from fresh and cadaveric biopsies [47]. We believe that further development of this technology could allow for the expansion of the inclusion criteria to cadaveric limbs with prolonged ischemia times. In addition to its expected applications for soft tissue reconstruction, this platform could theoretically allow for the development of complex bioengineered composite tissues, with enhanced cell-cell and cell-biomaterial interactions.

## Supporting information

S1 Movie Three-dimensional reconstruction of the contrast-enhanced computer tomography scan confirming perfusion of the acellular limb. The video shows the three-dimensional reconstruction obtained through the contrast-enhanced computerized tomography scans presented in Fig 1D.
(MOV)

## References

[1] Guyette JP, Gilpin SE, Charest JM, Tapias LF, Ren X, Ott HC. Perfusion decellularization of whole organs. Nature protocols. 2014;9(6):1451–68. doi: 10.1038/nprot.2014.097 . 24874812 10.1038/nprot.2014.097

[2] Ott HC, Matthiesen TS, Goh SK, Black LD, Kren SM, Netoff TI, et al Perfusion-decellularized matrix: using nature's platform to engineer a bioartificial heart. Nature medicine. 2008;14(2):213–21. doi: 10.1038/nm1684 . 18193059 10.1038/nm1684

[3] Ott HC, Clippinger B, Conrad C, Schuetz C, Pomerantseva I, Ikonomou L, et al Regeneration and orthotopic transplantation of a bioartificial lung. Nature medicine. 2010;16(8):927–33. doi: 10.1038/nm.2193 . 20628374 10.1038/nm.2193

[4] Gilpin SE, Guyette JP, Gonzalez G, Ren X, Asara JM, Mathisen DJ, et al Perfusion decellularization of human and porcine lungs: bringing the matrix to clinical scale. The Journal of heart and lung transplantation: the official publication of the International Society for Heart Transplantation. 2014;33(3):298–308. doi: 10.1016/j.healun.2013.10.030 . 24365767 10.1016/j.healun.2013.10.030

[5] Guyette JP, Charest JM, Mills RW, Jank BJ, Moser PT, Gilpin SE, et al Bioengineering Human Myocardium on Native Extracellular Matrix. Circulation research. 2016;118(1):56–72. doi: 10.1161/CIRCRESAHA.115.306874 . 26503464 10.1161/CIRCRESAHA.115.306874 PMC4740234

[6] Song JJ, Guyette JP, Gilpin SE, Gonzalez G, Vacanti JP, Ott HC. Regeneration and experimental orthotopic transplantation of a bioengineered kidney. Nature medicine. 2013;19(5):646–51. doi: 10.1038/nm.3154 . 23584091 10.1038/nm.3154 PMC3650107

[7] Mazza G, Rombouts K, Rennie Hall A, Urbani L, Vinh Luong T, Al-Akkad W, et al Decellularized human liver as a natural 3D-scaffold for liver bioengineering and transplantation. Scientific reports. 2015;5:13079 doi: 10.1038/srep13079 . 26248878 10.1038/srep13079 PMC4528226

[8] Peloso A, Urbani L, Cravedi P, Katari R, Maghsoudlou P, Fallas ME, et al The Human Pancreas as a Source of Protolerogenic Extracellular Matrix Scaffold for a New-generation Bioartificial Endocrine Pancreas. Annals of surgery. 2016;264(1):169–79. doi: 10.1097/SLA.0000000000001364 . 26649588 10.1097/SLA.0000000000001364 PMC4882269

[9] Ren X, Moser PT, Gilpin SE, Okamoto T, Wu T, Tapias LF, et al Engineering pulmonary vasculature in decellularized rat and human lungs. Nature biotechnology. 2015;33(10):1097–102. doi: 10.1038/nbt.3354 . 26368048 10.1038/nbt.3354

[10] Zhou H, Kitano K, Ren X, Rajab TK, Wu M, Gilpin SE, et al Bioengineering Human Lung Grafts on Porcine Matrix. Annals of surgery. 2017 doi: 10.1097/SLA.0000000000002129 . 28085694 10.1097/SLA.0000000000002129

[11] Jank BJ, Xiong L, Moser PT, Guyette JP, Ren X, Cetrulo CL, et al Engineered composite tissue as a bioartificial limb graft. Biomaterials. 2015;61:246–56. doi: 10.1016/j.biomaterials.2015.04.051 . 26004237 10.1016/j.biomaterials.2015.04.051 PMC4568187

[12] ASPS ASoPS. 2016 Plastic surgery statistics report. 2017.

[13] Grogan BF, Hsu JR, Skeletal Trauma Research C. Volumetric muscle loss. The Journal of the American Academy of Orthopaedic Surgeons. 2011;19 Suppl 1:S35–7. . 21304045 10.5435/00124635-201102001-00007

[14] Schaverien MV, Hart AM. Free muscle flaps for reconstruction of upper limb defects. Hand clinics. 2014;30(2):165–83, v–vi. doi: 10.1016/j.hcl.2014.01.001 . 24731608 10.1016/j.hcl.2014.01.001

[15] Klebuc M, Menn Z. Muscle flaps and their role in limb salvage. Methodist DeBakey cardiovascular journal. 2013;9(2):95–9. . 23805342 10.14797/mdcj-9-2-95 PMC3693523

[16] Lee SH, An SJ, Kim NR, Kim UJ, Kim JI. Reconstruction of Postburn Contracture of the Forefoot Using the Anterolateral Thigh Flap. Clinics in orthopedic surgery. 2016;8(4):444–51. doi: 10.4055/cios.2016.8.4.444 . 27904728 10.4055/cios.2016.8.4.444 PMC5114258

[17] Mast BA, Newton ED. Aggressive use of free flaps in children for burn scar contractures and other soft-tissue deficits. Annals of plastic surgery. 1996;36(6):569–75. . 8792964 10.1097/00000637-199606000-00002

[18] Brewer MB, Ochoa CJ, Woo K, Wartman SM, Nikolian V, Han S, et al Sartorius Muscle Flaps for Vascular Groin Wound Complications. The American surgeon. 2015;81(11):1163–9. . 26672588

[19] Agostini T, Lazzeri D, Spinelli G. Anterolateral thigh flap: systematic literature review of specific donor-site complications and their management. Journal of cranio-maxillo-facial surgery: official publication of the European Association for Cranio-Maxillo-Facial Surgery. 2013;41(1):15–21. doi: 10.1016/j.jcms.2012.05.003 . 22727900 10.1016/j.jcms.2012.05.003

[20] MacKenzie EJ, Bosse MJ, Kellam JF, Burgess AR, Webb LX, Swiontkowski MF, et al Factors influencing the decision to amputate or reconstruct after high-energy lower extremity trauma. The Journal of trauma. 2002;52(4):641–9. . 11956376 10.1097/00005373-200204000-00005

[21] Madden L, Juhas M, Kraus WE, Truskey GA, Bursac N. Bioengineered human myobundles mimic clinical responses of skeletal muscle to drugs. eLife. 2015;4:e04885 doi: 10.7554/eLife.04885 . 25575180 10.7554/eLife.04885 PMC4337710

[22] Chaturvedi V, Naskar D, Kinnear BF, Grenik E, Dye DE, Grounds MD, et al Silk fibroin scaffolds with muscle-like elasticity support in vitro differentiation of human skeletal muscle cells. Journal of tissue engineering and regenerative medicine. 2016 doi: 10.1002/term.2227 . 27878977 10.1002/term.2227 PMC5724504

[23] Fuoco C, Rizzi R, Biondo A, Longa E, Mascaro A, Shapira-Schweitzer K, et al In vivo generation of a mature and functional artificial skeletal muscle. EMBO molecular medicine. 2015;7(4):411–22. doi: 10.15252/emmm.201404062 . 25715804 10.15252/emmm.201404062 PMC4403043

[24] Chiron S, Tomczak C, Duperray A, Laine J, Bonne G, Eder A, et al Complex interactions between human myoblasts and the surrounding 3D fibrin-based matrix. PloS one. 2012;7(4):e36173 doi: 10.1371/journal.pone.0036173 . 22558372 10.1371/journal.pone.0036173 PMC3338613

[25] Juhas M, Ye J, Bursac N. Design, evaluation, and application of engineered skeletal muscle. Methods. 2016;99:81–90. doi: 10.1016/j.ymeth.2015.10.002 . 26455485 10.1016/j.ymeth.2015.10.002 PMC4821818

[26] Perniconi B, Coletti D. Skeletal muscle tissue engineering: best bet or black beast? Frontiers in physiology. 2014;5:255 doi: 10.3389/fphys.2014.00255 . 25071600 10.3389/fphys.2014.00255 PMC4082300

[27] Cornwell KG, Landsman A, James KS. Extracellular matrix biomaterials for soft tissue repair. Clinics in podiatric medicine and surgery. 2009;26(4):507–23. doi: 10.1016/j.cpm.2009.08.001 . 19778685 10.1016/j.cpm.2009.08.001

[28] Sicari BM, Rubin JP, Dearth CL, Wolf MT, Ambrosio F, Boninger M, et al An acellular biologic scaffold promotes skeletal muscle formation in mice and humans with volumetric muscle loss. Science translational medicine. 2014;6(234):234ra58 doi: 10.1126/scitranslmed.3008085 . 24786326 10.1126/scitranslmed.3008085 PMC5942588

[29] Mase VJ Jr., Hsu JR, Wolf SE, Wenke JC, Baer DG, Owens J, et al Clinical application of an acellular biologic scaffold for surgical repair of a large, traumatic quadriceps femoris muscle defect. Orthopedics. 2010;33(7):511 . 20608620 10.3928/01477447-20100526-24

[30] Corona BT, Wu X, Ward CL, McDaniel JS, Rathbone CR, Walters TJ. The promotion of a functional fibrosis in skeletal muscle with volumetric muscle loss injury following the transplantation of muscle-ECM. Biomaterials. 2013;34(13):3324–35. doi: 10.1016/j.biomaterials.2013.01.061 . 23384793 10.1016/j.biomaterials.2013.01.061

[31] Garg K, Ward CL, Rathbone CR, Corona BT. Transplantation of devitalized muscle scaffolds is insufficient for appreciable de novo muscle fiber regeneration after volumetric muscle loss injury. Cell and tissue research. 2014;358(3):857–73. doi: 10.1007/s00441-014-2006-6 . 25300647 10.1007/s00441-014-2006-6

[32] Dziki J, Badylak S, Yabroudi M, Sicari B, Ambrosio F, Stearns K, et al An acellular biologic scaffold treatment for volumetric muscle loss: results of a 13-patient cohort study. npj Regenerative Medicine. 2016;1:16008 doi: 10.1038/npjregenmed.2016.8 29302336 10.1038/npjregenmed.2016.8 PMC5744714

[33] Corona BT, Greising SM. Challenges to acellular biological scaffold mediated skeletal muscle tissue regeneration. Biomaterials. 2016;104:238–46. doi: 10.1016/j.biomaterials.2016.07.020 . 27472161 10.1016/j.biomaterials.2016.07.020

[34] Crapo PM, Gilbert TW, Badylak SF. An overview of tissue and whole organ decellularization processes. Biomaterials. 2011;32(12):3233–43. doi: 10.1016/j.biomaterials.2011.01.057 . 21296410 10.1016/j.biomaterials.2011.01.057 PMC3084613

[35] Jank BJ, Goverman J, Guyette JP, Charest JM, Randolph MA, Gaudette GR, et al Creation of a Bioengineered Skin Flap Scaffold with a Perfusable Vascular Pedicle. Tissue engineering Part A. 2017 doi: 10.1089/ten.TEA.2016.0487 . 28323545 10.1089/ten.tea.2016.0487 PMC5549829

[36] Gilbert TW, Sellaro TL, Badylak SF. Decellularization of tissues and organs. Biomaterials. 2006;27(19):3675–83. doi: 10.1016/j.biomaterials.2006.02.014 . 16519932 10.1016/j.biomaterials.2006.02.014

[37] Siemionow MZ, Rampazzo A, Gharb BB. Addressing religious and cultural differences in views on transplantation, including composite tissue allotransplantation. Annals of plastic surgery. 2011;66(4):410–5. doi: 10.1097/SAP.0b013e3182121db9 . 21372665 10.1097/SAP.0b013e3182121db9

[38] McDiarmid SV, Azari KK. Donor-related issues in hand transplantation. Hand clinics. 2011;27(4):545–52, x–xi. doi: 10.1016/j.hcl.2011.08.007 . 22051395 10.1016/j.hcl.2011.08.007

[39] Faulk DM, Carruthers CA, Warner HJ, Kramer CR, Reing JE, Zhang L, et al The effect of detergents on the basement membrane complex of a biologic scaffold material. Acta biomaterialia. 2014;10(1):183–93. doi: 10.1016/j.actbio.2013.09.006 . 24055455 10.1016/j.actbio.2013.09.006 PMC3857635

[40] Hwang J, San BH, Turner NJ, White LJ, Faulk DM, Badylak SF, et al Molecular assessment of collagen denaturation in decellularized tissues using a collagen hybridizing peptide. Acta biomaterialia. 2017;53:268–78. doi: 10.1016/j.actbio.2017.01.079 . 28161576 10.1016/j.actbio.2017.01.079 PMC5462463

[41] Ko SH, Bandyk DF, Hodgkiss-Harlow KD, Barleben A, Lane J 3rd. Estimation of brachial artery volume flow by duplex ultrasound imaging predicts dialysis access maturation. Journal of vascular surgery. 2015;61(6):1521–7. doi: 10.1016/j.jvs.2015.01.036 . 25769390 10.1016/j.jvs.2015.01.036

[42] Charest JM, Okamoto T, Kitano K, Yasuda A, Gilpin SE, Mathisen DJ, et al Design and validation of a clinical-scale bioreactor for long-term isolated lung culture. Biomaterials. 2015;52:79–87. doi: 10.1016/j.biomaterials.2015.02.016 . 25818415 10.1016/j.biomaterials.2015.02.016 PMC4568551

[43] Gilpin A, Yang Y. Decellularization Strategies for Regenerative Medicine: From Processing Techniques to Applications. BioMed research international. 2017;2017:9831534 doi: 10.1155/2017/9831534 . 28540307 10.1155/2017/9831534 PMC5429943

[44] Urbani L, Maghsoudlou P, Milan A, Menikou M, Hagen CK, Totonelli G, et al Long-term cryopreservation of decellularised oesophagi for tissue engineering clinical application. PloS one. 2017;12(6):e0179341 doi: 10.1371/journal.pone.0179341 . 28599006 10.1371/journal.pone.0179341 PMC5466304

[45] Piccoli M, Urbani L, Alvarez-Fallas ME, Franzin C, Dedja A, Bertin E, et al Improvement of diaphragmatic performance through orthotopic application of decellularized extracellular matrix patch. Biomaterials. 2016;74:245–55. doi: 10.1016/j.biomaterials.2015.10.005 . 26461117 10.1016/j.biomaterials.2015.10.005

[46] Zhang J, Hu ZQ, Turner NJ, Teng SF, Cheng WY, Zhou HY, et al Perfusion-decellularized skeletal muscle as a three-dimensional scaffold with a vascular network template. Biomaterials. 2016;89:114–26. doi: 10.1016/j.biomaterials.2016.02.040 . 26963901 10.1016/j.biomaterials.2016.02.040

[47] Porzionato A, Sfriso MM, Pontini A, Macchi V, Petrelli L, Pavan PG, et al Decellularized Human Skeletal Muscle as Biologic Scaffold for Reconstructive Surgery. International journal of molecular sciences. 2015;16(7):14808–31. doi: 10.3390/ijms160714808 . 26140375 10.3390/ijms160714808 PMC4519873

[48] Duisit J, Maistriaux L, Taddeo A, Orlando G, Joris V, Coche E, et al Bioengineering a Human Face Graft: The Matrix of Identity. Annals of surgery. 2017;266(5):754–64. doi: 10.1097/SLA.0000000000002396 . 28742686 10.1097/SLA.0000000000002396

[49] Teodori L, Costa A, Marzio R, Perniconi B, Coletti D, Adamo S, et al Native extracellular matrix: a new scaffolding platform for repair of damaged muscle. Frontiers in physiology. 2014;5:218 doi: 10.3389/fphys.2014.00218 . 24982637 10.3389/fphys.2014.00218 PMC4058757

[50] Badylak SF, Taylor D, Uygun K. Whole-organ tissue engineering: decellularization and recellularization of three-dimensional matrix scaffolds. Annual review of biomedical engineering. 2011;13:27–53. doi: 10.1146/annurev-bioeng-071910-124743 . 21417722 10.1146/annurev-bioeng-071910-124743 PMC10887492

[51] Freedman BR, Bade ND, Riggin CN, Zhang S, Haines PG, Ong KL, et al The (dys)functional extracellular matrix. Biochimica et biophysica acta. 2015;1853(11 Pt B):3153–64. doi: 10.1016/j.bbamcr.2015.04.015 . 25930943 10.1016/j.bbamcr.2015.04.015 PMC4846286

[52] Hajimiri M, Shahverdi S, Kamalinia G, Dinarvand R. Growth factor conjugation: strategies and applications. Journal of biomedical materials research Part A. 2015;103(2):819–38. doi: 10.1002/jbm.a.35193 . 24733811 10.1002/jbm.a.35193

[53] Dimitrievska S, Cai C, Weyers A, Balestrini JL, Lin T, Sundaram S, et al Click-coated, heparinized, decellularized vascular grafts. Acta biomaterialia. 2015;13:177–87. doi: 10.1016/j.actbio.2014.11.015 . 25463496 10.1016/j.actbio.2014.11.015 PMC4293247

[54] Kitano K, Schwartz DM, Zhou H, Gilpin SE, Wojtkiewicz GR, Ren X, et al Bioengineering of functional human induced pluripotent stem cell-derived intestinal grafts. Nature communications. 2017;8(1):765 doi: 10.1038/s41467-017-00779-y . 29018244 10.1038/s41467-017-00779-y PMC5635127

[55] Werner NL, Alghanem F, Rakestraw SL, Sarver DC, Nicely B, Pietroski RE, et al Ex Situ Perfusion of Human Limb Allografts for 24 Hours. Transplantation. 2017;101(3):e68–e74. doi: 10.1097/TP.0000000000001500 . 28222055 10.1097/TP.0000000000001500

[56] Shandalov Y, Egozi D, Koffler J, Dado-Rosenfeld D, Ben-Shimol D, Freiman A, et al An engineered muscle flap for reconstruction of large soft tissue defects. Proceedings of the National Academy of Sciences of the United States of America. 2014;111(16):6010–5. doi: 10.1073/pnas.1402679111 . 24711414 10.1073/pnas.1402679111 PMC4000846

[57] Richard PL, Gosselin C, Laliberte T, Paradis M, Goulet M, Tremblay JP, et al A first semimanual device for clinical intramuscular repetitive cell injections. Cell transplantation. 2010;19(1):67–78. . 20370989 10.3727/096368909X478812

[58] Dellavalle A, Sampaolesi M, Tonlorenzi R, Tagliafico E, Sacchetti B, Perani L, et al Pericytes of human skeletal muscle are myogenic precursors distinct from satellite cells. Nat Cell Biol. 2007;9(3):255–67. doi: 10.1038/ncb1542 . 17293855 10.1038/ncb1542

[59] Cossu G, Previtali SC, Napolitano S, Cicalese MP, Tedesco FS, Nicastro F, et al Intra-arterial transplantation of HLA-matched donor mesoangioblasts in Duchenne muscular dystrophy. EMBO molecular medicine. 2015;7(12):1513–28. doi: 10.15252/emmm.201505636 . 26543057 10.15252/emmm.201505636 PMC4693504

[60] Tedesco FS, Hoshiya H, D'Antona G, Gerli MF, Messina G, Antonini S, et al Stem cell-mediated transfer of a human artificial chromosome ameliorates muscular dystrophy. Science translational medicine. 2011;3(96):96ra78 Epub 2011/08/19. doi: 10.1126/scitranslmed.3002342 . 21849666 10.1126/scitranslmed.3002342

[61] Hitchcock T, Niklason L. Lymphatic tissue engineering: progress and prospects. Annals of the New York Academy of Sciences. 2008;1131:44–9. doi: 10.1196/annals.1413.004 . 18519958 10.1196/annals.1413.004 PMC2610296

[62] Marino D, Luginbuhl J, Scola S, Meuli M, Reichmann E. Bioengineering dermo-epidermal skin grafts with blood and lymphatic capillaries. Science translational medicine. 2014;6(221):221ra14 doi: 10.1126/scitranslmed.3006894 . 24477001 10.1126/scitranslmed.3006894

